# Ammonia as an Antidote for Belladonna

**Published:** 1841-11-20

**Authors:** T. L. Walker

**Affiliations:** Blockley Hospital


To the Editors of the Medical Examiner.

### *Ammonia as an Antidote for Belladonna.*

To the Editors of the Medical Examiner.

GENTLEMEN,—A curious case recently occurred, in one of the wards of this Hospital, the result of which I disclose, as it may furnish a useful fact relative to the administration of large quantities of belladonna.

Elizabeth Dale, a coloured woman, æt. 31, was admitted October the 3d, labouring under a slight catarrh. During the afternoon of her entrance, such medicines as her situation seemed to indicate, were ordered; but, from the negligence of her nurse, a prescription intended as an external application for another patient was administered to her; viz:

℞ Extract. Belladonnæ, ʒj.

Lin. Camphoræ comp., ℥vj. M.

Of this she took *one ounce*, containing *ten grains* of the extract, on the evening of the third; and, no unpleasant effects following, she passed a comfortable night. On awaking, she thought her cold much relieved, and, as directed by the nurse, swallowed an additional half ounce of the compound, or five grains of the extract. This had remained but a short time upon her stomach, when it was returned by laboured vomiting. After the lapse of half an hour, the dose being repeated, was again returned almost immediately. The emesis following the two last doses most probably resulted from the irritable condition of the stomach induced by the draught of the previous evening. Thus, taking it for granted that the extract and liniment were thoroughly combined, it is seen that *twenty grains* of this powerful narcotic poison were taken into the stomach, ten grains being retained for *twelve hours*, without the patient's experiencing any disagreeable symptoms. The next morning, however, when she was visited, some slight influence of the narcotic was discovered, though not sufficiently marked, of itself, to attract attention, had not inquiry been directed by a discovery of the mistake. There was some slight dryness of the fauces, but no effect upon the brain or pupil could be detected. Immediately on ascertaining what she had taken, copious draughts of warm mucilage were directed, though little could be expected from this precautionary measure, as narcotic poisons usually exert their influence very speedily.

Could the *ammonia* have acted as an antidote in this case? It may be suspected that the extract used was of inferior quality; but on being subsequently tested, it has proved itself amply efficient. From a variety of experiments, M. Runge asserts that the alkaline solutions render belladonna inert.

T. L. WALKER, M. D.
*Blockley Hospital*, Nov. 22, 1841.

